# Dynamic proprioceptive training improves functional recovery in sprinters with patellofemoral pain: A randomized trial

**DOI:** 10.1371/journal.pone.0353107

**Published:** 2026-07-24

**Authors:** Devadharshini B, Shenbaga Sundaram Subramanian, Jeevarathinam Thirumalai, Hadeel R. Bakhsh, Saad Suleman Alfawaz, Rayan Jastania, Riziq Allah Mustafa Gaowgzeh, Thamer A. Altaim, Fadwa Alhalaiqa, Porkodi Arjunan, Yasmeen Imtiaz G

**Affiliations:** 1 Saveetha College of Physiotherapy, Saveetha Institute of Medical and Technical Sciences (SIMATS), Chennai, Tamil Nadu, India; 2 Department of Rehabilitation Sciences, College of Health and Rehabilitation Sciences, Princess Nourah bint Abdulrahman University, Riyadh, Saudi Arabia; 3 Department of Physical Therapy, Faculty of Medical Rehabilitation Sciences, King Abdulaziz University, Jeddah, Saudi Arabia; 4 Department of Physical Therapy, Faculty of Applied Medical Sciences, The Hashemite University, Zarqa, Jordan; 5 College of Nursing, Qatar University, Doha, Qatar; 6 Nursing Department, College of Applied Medical Sciences, King Faisal University, Hofuf, Saudi Arabia; 7 SRM College of Physiotherapy, Faculty of Medicine and Health Sciences, SRM Institute of Science and Technology, Chengalpattu, Tamil Nadu, India; Shahrood University of Technology, IRAN, ISLAMIC REPUBLIC OF

## Abstract

**Background:**

Patellofemoral pain syndrome (PFPS) is a common musculoskeletal condition among physically active individuals, particularly sprinters, and is frequently associated with pain, impaired balance, and reduced functional performance. This study compared the effects of dynamic proprioceptive training and conventional strengthening on multidomain functional recovery in recreational sprinters with PFPS.

**Methods:**

A two-arm, assessor-blinded randomized controlled trial was conducted among 60 recreational sprinters with unilateral PFPS. Participants were randomly allocated to either a Dynamic Proprioceptive Training (DPT) group or a Strengthening Program (SP) group (n = 30 each). Both groups received supervised rehabilitation three times weekly for 12 weeks. Primary outcomes included pain intensity (Numeric Pain Rating Scale [NPRS]), dynamic balance (Y-Balance Test [YBT]), and single-hop distance. Secondary outcomes included limb symmetry index (LSI), recovery rates, and a composite Recovery Efficiency Index (REI). Assessments were performed at baseline, 6 weeks, and 12 weeks. Repeated-measures ANOVA and regression-based analyses were performed. This trial was registered with the Clinical Trials Registry of India (CTRI/2025/07/090253).

**Results:**

Both groups improved significantly over time (p < 0.001). However, the DPT group demonstrated greater clinically meaningful improvements than the SP group. At 12 weeks, between-group differences favored DPT for pain reduction (MD = 1.01 NPRS points, 95% CI: 0.61–1.41), dynamic balance (MD = 5.73 cm, 95% CI: 4.31–7.15), and hop performance (MD = 35.84 mm, 95% CI: 16.84–54.85).

**Conclusion:**

Dynamic proprioceptive training produced greater multidomain functional recovery than conventional strengthening in recreational sprinters with PFPS. Incorporating sensorimotor and perturbation-based exercises into rehabilitation programmes may improve functional recovery, limb symmetry, and sport-specific performance in athletic populations.

## Introduction

Patellofemoral pain syndrome (PFPS) is one of the most common musculoskeletal conditions affecting physically active individuals, particularly running and sprinting athletes [1]. It is characterized by anterior or retropatellar knee pain that is aggravated by activities involving increased patellofemoral joint loading, such as squatting, stair negotiation, prolonged sitting, and running. Beyond its high prevalence in athletic populations, PFPS is also associated with substantial healthcare utilization, rehabilitation expenses, reduced sports participation, and productivity loss resulting from persistent pain and functional limitations [2,3]. PFPS accounts for approximately 25–40% of knee-related complaints encountered in sports medicine and rehabilitation settings, highlighting its substantial clinical and athletic relevance [4,5].

The etiology of PFPS is multifactorial and involves complex interactions between biomechanical, neuromuscular, and tissue-level factors. Deficits in proximal and local neuromuscular control are frequently implicated in the development and persistence of symptoms [6–8]. Weakness or delayed activation of the hip abductors, hip external rotators, and quadriceps musculature—particularly the vastus medialis obliquus—may contribute to abnormal lower-limb kinematics during dynamic tasks [9–11]. These alterations can result in excessive femoral internal rotation, dynamic knee valgus, and patellar maltracking, ultimately increasing stress on the patellofemoral joint and contributing to pain and functional limitation [12–14].

In addition to muscular deficits, growing evidence suggests that impaired proprioception and dynamic postural control play an important role in PFPS [15,16]. Proprioception is essential for maintaining joint stability and coordinating neuromuscular responses during weight-bearing and high-velocity movements [17–19]. Individuals with PFPS have demonstrated reduced proprioceptive acuity, impaired balance performance, and altered neuromuscular activation patterns compared with asymptomatic individuals [20,21]. These sensorimotor impairments may compromise the ability to effectively control lower-limb alignment and attenuate forces during athletic activities such as sprinting [22,23].

Conventional rehabilitation strategies for PFPS have traditionally emphasized strengthening of the quadriceps, hip, and core musculature [24]. While strengthening programs are effective in reducing pain and improving functional outcomes, isolated strength training may not fully address the neuromuscular and sensorimotor deficits associated with PFPS [25,26]. As a result, contemporary rehabilitation approaches increasingly incorporate neuromuscular retraining and proprioceptive exercises aimed at improving dynamic joint stability and motor control [27,28]. Recent evidence has further suggested that multidimensional rehabilitation approaches incorporating sensorimotor and mindfulness-based strategies may improve pain and functional outcomes in individuals with patellofemoral pain syndrome, including physically active running populations, thereby supporting the importance of interventions extending beyond isolated strengthening paradigms.

Dynamic proprioceptive training represents a promising intervention strategy for addressing these deficits [28]. Such programs typically include exercises performed on unstable surfaces, perturbation-based tasks, and sport-specific movement challenges designed to enhance sensorimotor integration and dynamic postural control [28–30]. These interventions may promote improved neuromuscular coordination and functional stability, potentially leading to better rehabilitation outcomes for athletes with PFPS [29,31–33].

Previous randomized controlled trials have compared proprioceptive or sensorimotor training with strengthening-based rehabilitation in individuals with patellofemoral pain syndrome, including women and running populations [28,29]. However, no prior randomized controlled trial has specifically investigated the effects of dynamic proprioceptive training in recreational sprinters with PFPS, a population characterized by high sprint-related loading demands and rapid neuromuscular control requirements. Additionally, limited evidence exists regarding the multidimensional effects of such interventions on pain, dynamic balance, limb symmetry, and sport-specific functional performance outcomes in sprinters. The observed improvements in hop performance may be associated with enhanced sensorimotor integration, reactive neuromuscular control, and lower-limb joint stabilization during dynamic athletic tasks. Improved proprioceptive acuity and dynamic postural control may also be associated with more efficient force attenuation, movement coordination, and limb loading symmetry during single-leg functional activities commonly required in sprinting.

Therefore, the present randomized controlled trial aimed to compare the effects of a 12-week dynamic proprioceptive training program with a conventional strengthening program on pain intensity, dynamic balance, and single-hop functional performance in recreational sprinters with PFPS. It was hypothesized that dynamic proprioceptive training would result in greater improvements in pain reduction, dynamic balance, and functional performance compared with conventional strengthening, potentially through enhanced neuromuscular control and sensorimotor function.

## Materials and methods

### Ethical approval and trial registration

This study was approved by the Institutional Scientific Review Board (ISRB) 046/05/2025/ISRB/PGSR/SCPT of Saveetha College of Physiotherapy, Saveetha Institute of Medical and Technical Sciences (SIMATS), India, prior to the commencement of participant recruitment, in accordance with the ethical principles outlined in the Declaration of Helsinki (World Medical Association, 2013). The trial was prospectively registered with the Clinical Trials Registry-India (CTRI/2025/07/090253), a primary registry of the World Health Organization’s International Clinical Trials Registry Platform. Participant recruitment for this randomized controlled trial was conducted from 30 July 2025 to 7 February 2026 (https://ctri.nic.in/Clinicaltrials/pmaindet2.php?EncHid=MTM0ODg4&Enc=&userName=). All participants were adults (≥18 years) and provided written informed consent prior to enrollment after receiving a detailed explanation of the study objectives, procedures, potential risks, benefits, and the right to withdraw at any stage without penalty. Written informed consent for publication of the participant images presented in this manuscript was obtained from the participant in accordance with the PLOS consent form. Data confidentiality and participant anonymity were maintained throughout the study in compliance with institutional data protection guidelines.

### Study design

This study was designed as a two-arm, parallel-group, assessor-blinded, randomized controlled trial (RCT) conducted in accordance with the Consolidated Standards of Reporting Trials (CONSORT) 2010 guidelines. The study followed a pre-test-post-test experimental design with three assessment timepoints: baseline (T0), 6 weeks (T1), and 12 weeks (T2). Participants were randomly allocated to either the DPT group or SP group using computer-generated block randomization with a fixed block size of four. The random allocation sequence was generated using Random Allocation Software version 2.0 (Isfahan University of Medical Sciences, Isfahan, Iran) by an independent researcher not involved in recruitment, assessment, or intervention delivery.

No post hoc modifications were made to the study protocol, intervention procedures, outcome measures, eligibility criteria, or statistical analysis plan after participant enrollment and trial commencement.

### Study setting

The study was conducted at the outpatient physiotherapy rehabilitation unit affiliated with Saveetha College of Physiotherapy, Saveetha Institute of Medical and Technical Sciences, Chennai, India, between August 2025 and December 2025. All assessments and intervention sessions were carried out in a climate-controlled laboratory environment equipped with standardized testing apparatus. The facility included a dedicated biomechanics assessment area with non-slip flooring for balance and hop testing, ensuring participant safety and measurement consistency across all evaluation sessions.

### Participants

**Fig 1:**
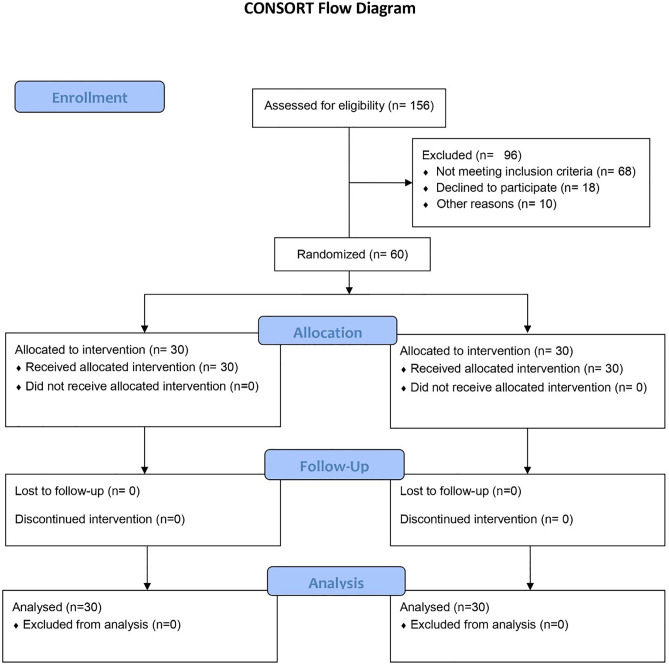
CONSORT flow diagram of participant recruitment, allocation, follow-up, and analysis. A total of 156 individuals were assessed for eligibility, of whom 96 were excluded. Sixty participants were randomized into the Dynamic Proprioceptive Training (DPT) and Strengthening Program (SP) groups (n = 30 each). All participants received the allocated intervention and were included in the final analysis.

A total of 156 individuals were assessed for eligibility, of whom 96 were excluded (68 did not meet the inclusion criteria, 18 declined to participate, and 10 were excluded due to logistical constraints). Sixty eligible participants were subsequently randomized into two groups (n = 30 per group) (Fig 1). All randomized participants received the allocated intervention, completed the study protocol, and were included in the final analysis.

Participants comprised recreational sprinters diagnosed with unilateral patellofemoral pain syndrome (PFPS), recruited from local athletics clubs, university sports teams, and community sports rehabilitation centres through convenience sampling supplemented by advertisement-based recruitment. Recreational sprinters were defined as individuals who engaged in sprint-based training or competition at a sub-elite level for at least three sessions per week over the preceding six months.

### Eligibility criteria

The following inclusion criteria were applied: (1) age between 18 and 30 years; (2) clinically confirmed diagnosis of unilateral PFPS based on the presence of anterior or retropatellar knee pain of insidious onset, aggravated by at least two of the following activities: squatting, stair climbing, prolonged sitting, running, or kneeling (Crossley et al., 2016); (3) both male and female recreational sprinters who satisfied the eligibility criteria were considered eligible for inclusion in the study; (4) symptom duration of at least four weeks; (5) pain intensity of ≥3 on the Numeric Pain Rating Scale (NPRS) during at least one provocative activity; (6) active participation in sprint-based athletics for ≥6 months prior to symptom onset; and (7) willingness to comply with the 12-week intervention protocol and attend all scheduled assessment sessions.

The exclusion criteria were: (1) history of knee surgery or intra-articular injection within the preceding 12 months; (2) concomitant ligamentous injury (anterior cruciate ligament, posterior cruciate ligament, or collateral ligament pathology) confirmed by clinical examination or imaging; (3) meniscal tear or other internal derangement of the knee; (4) patellar instability or recurrent subluxation or dislocation; (5) inflammatory arthropathy, systemic connective tissue disorder, or neurological condition affecting lower limb function; (6) lower limb fracture within the preceding 12 months; (7) concurrent participation in any other structured rehabilitation or physical therapy programme; and (8) use of corticosteroid or analgesic medications within two weeks prior to enrolment.

### Sample size estimation

An a priori sample size calculation was performed by an independent biostatistician using G*Power software (version 3.1.9.7; Heinrich-Heine-Universität Düsseldorf, Germany) for a repeated-measures ANOVA evaluating the group × time interaction effect. The study was primarily powered based on the primary outcome of pain intensity measured using the Numeric Pain Rating Scale (NPRS), as pain reduction represented the principal clinical endpoint of interest.

The sample size estimation assumed a clinically meaningful between-group difference of 2 NPRS points with an estimated standard deviation of 1.73 derived from previous patellofemoral pain literature. Using a two-tailed α error probability of 0.05, statistical power (1 − β) of 0.80, two groups, and three repeated measurements, with an assumed correlation among repeated measures of 0.50 and nonsphericity correction ε = 1, the estimated minimum required sample size was 44 participants. To account for potential attrition of up to 25%, the target sample size was increased to 60 participants (30 per group).

### Randomisation and allocation concealment

Participants who met all eligibility criteria and provided written informed consent were randomly allocated to the DPT group (n = 30) or the SP group (n = 30) using a computer-generated block randomisation sequence with a fixed block size of four, created by an independent statistician who was not involved in participant recruitment or assessment. The allocation sequence was concealed using sequentially numbered, opaque, sealed envelopes. Each envelope was opened only after the participant had completed the baseline assessment, thereby ensuring that the assessor and the participant remained unaware of group allocation at the time of initial evaluation. Stratification was performed by gender and affected side to ensure balanced distribution of these prognostic variables across groups.

### Blinding

Given the nature of the physical rehabilitation interventions, blinding of participants and treating physiotherapists was not feasible. However, outcome assessors who performed all measurements at T0, T1, and T2 were blinded to group allocation. Assessors were trained in standardized measurement protocols and had no involvement in intervention delivery. The statistician responsible for the data analysis was also blinded to group allocation until completion of the primary analyses. Participants were instructed not to disclose their treatment allocation to the assessors during outcome assessment sessions.

### Interventions

Both intervention groups received supervised treatment sessions three times per week for 12 consecutive weeks, totaling 36 sessions. Each session lasted approximately 45–60 minutes and was administered by licensed physiotherapists with a minimum of three years of clinical experience in musculoskeletal rehabilitation. Progression criteria were standardized using a protocol manual before trial initiation. Advancement to higher exercise difficulty levels was permitted only when participants maintained pain below 3/10 on the Numeric Pain Rating Scale during exercise, demonstrated correct movement quality, stable postural control, and absence of compensatory movement patterns. All sessions were conducted on a one-to-one basis to ensure treatment fidelity and adherence. Participants in both groups were permitted to continue their routine daily activities but were instructed to refrain from any additional structured exercise or rehabilitation programme for the duration of the study. Additionally, the protocol was reviewed during therapist training workshops prior to study initiation to ensure treatment fidelity and consistency in progression across participants.

### Dynamic proprioceptive training (DPT) group

Participants allocated to the DPT group received a progressive, multicomponent dynamic proprioceptive training programme specifically designed for sprinters with PFPS. The programme targeted the restoration of neuromuscular control, dynamic joint stability, and sport-specific proprioceptive acuity through systematically graded exercises. The detailed week-by-week progression of exercises, loading parameters, and advancement criteria for both intervention groups is presented in Table 1. Each session comprised the following components:

**Table 1:** Progressive rehabilitation protocols and advancement criteria for the dynamic proprioceptive training and strengthening program groups across the 12-week intervention period.

Weeks	Dynamic Proprioceptive Training (DPT) Progression	Strengthening Program (SP) Progression	Progression Criteria
Weeks 1–2	Double-leg and single-leg balance on stable surface; foam-pad stance; low-level perturbation drills; 2–3 sets × 10 reps/30 sec hold	Isometric quadriceps sets, straight leg raises, mini squats, clamshells at 60% 1-RM; 3 sets × 10 reps	Pain maintained <3/10 NPRS, correct movement pattern, no post-exercise symptom aggravation
Weeks 3–4	Single-leg stance on foam with eyes closed; wobble board weight shifts; multidirectional reach tasks; 3 sets × 12 reps	Progression to resisted band walks, unilateral mini squats, seated knee extension at 65–70% 1-RM; 3 sets × 12 reps	Ability to complete all exercises with stable alignment and minimal compensatory movement
Weeks 5–6	Dynamic BOSU squats, reactive stepping drills, perturbation balance training; 3 sets × 12–15 reps	Increased resistance for hip abductors/external rotators; unilateral strengthening exercises at 70% 1-RM; 3 sets × 12 reps	Successful completion of previous level without pain increase or balance loss
Weeks 7–8	Single-leg hop-and-hold on unstable surfaces, multidirectional perturbation tasks, reactive agility drills; 3–4 sets × 15 reps	Advanced closed kinetic chain strengthening, lateral band walks with higher resistance, squat progression at 75% 1-RM	Maintenance of dynamic knee control and exercise tolerance during functional tasks
Weeks 9–10	Sport-specific bounding drills, acceleration–deceleration perturbation exercises, unstable landing mechanics; 4 sets × 15 reps	Functional strengthening with increased load and complexity, unilateral squat/lunge variations at 75–80% 1-RM	Completion of exercises with symmetrical movement and controlled landing mechanics
Weeks 11–12	High-level reactive neuromuscular drills, sprint-specific proprioceptive tasks, advanced perturbation and agility exercises; 4 sets × 15–20 reps	High-resistance functional strengthening and endurance-based repetitions at 80% 1-RM; 3–4 sets × 15 reps	Independent performance with proper neuromuscular control, pain-free functional execution, and readiness for sport-specific activity

(1) Warm-up phase (5–10 minutes): Low-intensity cycling or brisk walking on a treadmill at a self-selected comfortable pace to increase tissue temperature and neuromuscular readiness.
(2) Proprioceptive and neuromuscular training phase (30–40 minutes): This phase included exercises performed on progressively challenging unstable surfaces (foam pads, BOSU balls, wobble boards, and tilt boards). Specific exercises included: (a) single-leg stance with perturbation on foam pads with eyes open and eyes closed; (b) dynamic single-leg squats on BOSU balls; (c) multidirectional reach tasks on wobble boards mimicking sprint-specific lower limb loading patterns; (d) reactive stepping drills incorporating unanticipated directional cues; (e) single-leg hop-and-hold landings on compliant surfaces progressing to sport-specific bounding drills; and (f) perturbation-based agility exercises simulating sprint acceleration and deceleration phases. Volume and intensity were individually titrated based on each participant’s pain threshold (maintained below 3/10 on the NPRS during exercise) and performance capacity.
(3) Cool-down phase (5 minutes): Gentle stretching of the quadriceps, hamstrings, iliotibial band, and gastrocnemius-soleus complex, each held for 30 seconds with two repetitions.

### Strengthening program (SP) group

Participants in the SP group received a conventional progressive strengthening programme targeting the lower extremity musculature implicated in PFPS. Each session comprised:

(1) Warm-up phase (5–10 minutes): Identical to the DPT group.
(2) Strengthening phase (30–40 minutes): This phase comprised open and closed kinetic chain exercises targeting the quadriceps (with emphasis on the vastus medialis obliquus), hip abductors, hip external rotators, and core stabilisers. Specific exercises included: (a) isometric quadriceps sets at multiple angles (0°, 30°, 60°, and 90° of knee flexion); (b) straight leg raises in four directions (flexion, abduction, adduction, and extension); (c) seated knee extensions (terminal range, 90–45° to full extension); (d) bilateral and unilateral mini-squats (0–45° knee flexion range); (e) lateral band walks with resistance bands; (f) clamshell exercises with progressive resistance; and (g) prone hip extension exercises. Resistance was progressed systematically using the repetition maximum continuum, beginning at 60% of the estimated one-repetition maximum (1-RM) and advancing to 80% of 1-RM by the final phase. Exercises were performed using standardized rehabilitation equipment, including resistance bands, ankle weights, exercise mats, and adjustable treatment benches, with progression tailored according to participant tolerance and performance capacity. Participants performed 3 sets of 10–15 repetitions for each exercise with 60–90 seconds of rest between sets.
(3) Cool-down phase (5 minutes): Identical to the DPT group.

### Outcome measures

All outcome measures were assessed at three timepoints: baseline (T0), at 6 weeks (T1), and at 12 weeks post-intervention (T2). Assessments were performed by trained, blinded assessors using standardised protocols. The order of testing was consistent across all sessions to minimise the influence of fatigue: pain assessment was performed first, followed by dynamic balance testing, and finally the hop performance assessment after adequate rest.

#### Primary outcome measures.

Pain Intensity. Pain intensity was assessed using the Numeric Pain Rating Scale (NPRS), an 11-point ordinal scale ranging from 0 (“no pain”) to 10 (“worst imaginable pain”). Participants were asked to rate their current knee pain at rest and the worst pain experienced during aggravating activities in the preceding 24 hours. The higher of the two values was recorded as the pain score for analysis. The NPRS has demonstrated excellent test-retest reliability (ICC = 0.92–0.95) and a minimum clinically important difference (MCID) of 2 points in musculoskeletal pain populations (24).

Dynamic Balance. Dynamic balance of the injured lower limb was assessed using the Y-Balance Test (YBT), a reliable and validated instrument for evaluating dynamic postural control in athletes. The YBT requires the participant to maintain single-leg stance on the injured limb while reaching maximally in the anterior, posteromedial, and posterolateral directions with the contralateral limb. Three practice trials were permitted in each direction, followed by three recorded trials. The maximal reach distance (in centimetres) in each direction was normalised to limb length, and the composite score was calculated using the formula: Composite Score = [(Anterior + Posteromedial + Posterolateral)/ (3 × Limb Length)] × 100. The normalized composite Y-Balance score was selected as the primary analytical outcome because it provides an integrated measure of dynamic postural control while accounting for inter-individual differences in limb length and anthropometric variability. Composite normalization has been widely recommended in athletic and patellofemoral pain research to improve comparability across participants and reduce directional measurement variability. Accordingly, the composite score was prespecified as the primary balance outcome measure in the present study [34,35]. The YBT has demonstrated excellent intra-rater reliability (ICC = 0.85–0.91) and good discriminative validity for identifying lower extremity deficits in athletic populations [16].

Functional hop performance and limb symmetry were included because they represent clinically meaningful indicators of lower-extremity function in sprinters. Sprinting activities require rapid force generation, single-leg stability, efficient neuromuscular coordination, and symmetrical limb loading during acceleration, deceleration, and propulsion phases. Deficits in hop performance or limb symmetry may therefore reflect persistent functional limitations and altered movement strategies that could affect athletic performance and recovery in individuals with PFPS. The single-hop for distance test has demonstrated good-to-excellent within-day (ICC = 0.89–0.93) and between-day (ICC = 0.85–0.90) reliability in athletic populations [50].

#### Secondary outcome measures.

Limb Symmetry Index. The Limb Symmetry Index (LSI) was calculated as the ratio of the single-hop distance of the injured limb to that of the non-injured limb, expressed as a percentage: LSI (%) = (Injured Limb Hop Distance/ Non-Injured Limb Hop Distance) × 100. An LSI of ≥90% is generally considered the threshold for acceptable limb symmetry and readiness for return to sport [72]. LSI was calculated at baseline (T0) and at 12 weeks (T2).

Clinically meaningful improvement was predefined prior to analysis as a reduction of ≥2 points on the Numeric Pain Rating Scale (NPRS), consistent with established minimum clinically important difference thresholds for musculoskeletal pain populations, and achievement of a Limb Symmetry Index (LSI) ≥90%, indicating acceptable functional symmetry and return-to-sport readiness.

Recovery Rates. The rate of recovery for each primary outcome was calculated as the total change from baseline to 12 weeks divided by the intervention duration (12 weeks), yielding a weekly rate of change. Specifically, Rate of Pain Recovery (NPRS points/week) = (NPRST0 - NPRST2)/ 12; Rate of Balance Recovery (cm/week) = (YBTT2 - YBTT0)/ 12; and Rate of Hop Recovery (mm/week) = (HopT2 - HopT0)/ 12.

Recovery Efficiency Index. A composite Recovery Efficiency Index (REI) was derived to summarize multidimensional rehabilitation recovery into a single integrated metric. The rationale for constructing the REI was that recovery following patellofemoral pain rehabilitation is inherently multidimensional and involves concurrent changes in pain reduction, dynamic balance, and sport-specific functional performance rather than improvement within a single isolated domain alone. Reporting each outcome separately remains clinically important; however, separate analyses may not fully capture the overall efficiency and coordination of recovery across interacting functional domains.

The REI was therefore developed as an exploratory summary indicator to reflect each participant’s integrated recovery profile across the principal rehabilitation targets. The index was calculated as the unweighted sum of z-score standardized recovery rates for pain reduction, balance improvement, and hop performance improvement. Z-score standardization was used to place variables measured on different numerical scales (NPRS points, centimetres, and millimetres) onto a common metric and to prevent disproportionate influence of outcomes with larger absolute numerical ranges. An unweighted approach was selected because no validated evidence currently exists to support differential weighting of these domains in recreational sprinters with PFPS.

Importantly, the REI was not intended to replace interpretation of the individual clinical outcomes, which were reported separately as the primary analyses of the study. Rather, the index was used as an exploratory adjunct measure to characterize the overall multidomain recovery trajectory and facilitate integrated interpretation of rehabilitation response patterns. Because the REI has not yet undergone external validation or psychometric testing, it should be interpreted cautiously and considered hypothesis-generating.

### Standardization of testing procedures

To minimize measurement variability and ensure reproducibility, all testing procedures were standardized as follows: (1) assessments were conducted at the same time of day (±2 hours) for each participant to control for diurnal variation; (2) participants wore standardized athletic footwear and clothing during all assessments; (3) a five-minute warm-up on a stationary cycle ergometer at a self-selected comfortable intensity preceded all functional tests; (4) standardized verbal instructions were read from a script prior to each test to ensure consistency; (5) the same equipment (measuring tapes, Y-Balance test kit, and NPRS form) was used across all timepoints; and (6) participants were instructed to refrain from vigorous physical activity for 24 hours prior to each assessment session.

### Treatment adherence and fidelity

Adherence to the intervention protocol was monitored using session attendance logs maintained by the treating physiotherapists. A minimum attendance threshold of 80% (29 out of 36 sessions) was required for inclusion in the per-protocol analysis. Treatment fidelity was ensured through: (1) a detailed, standardised intervention manual provided to all treating physiotherapists; (2) a two-day training workshop conducted prior to the commencement of the trial to ensure uniformity in intervention delivery; and (3) periodic audits of session logs and exercise prescription records by the principal investigator. Any adverse events or symptom exacerbations occurring during the intervention period were documented using a standardised adverse event reporting form.

### Statistical analysis

All statistical analyses were performed using R (version 4.3.0; R Foundation for Statistical Computing, Vienna, Austria) with the rstatix and ez packages. The significance level was set at α = 0.05 for all analyses. Descriptive statistics were computed as means ± standard deviations (SD) for continuous variables and frequencies with percentages for categorical variables.

Missing data handling. Missing outcome data and participant attrition were assessed prior to statistical analysis. No participant withdrawals, missing outcome measurements, or protocol deviations occurred during the study period; therefore, complete-case analyses were performed. The intention-to-treat and per-protocol datasets were consequently identical. Although no imputation procedures were ultimately required, multiple imputation methods had been prespecified in the statistical analysis plan for use if missing outcome data exceeded minimal levels.

Multiple comparison adjustment. Because the primary hypotheses and outcome measures were predefined a priori and the repeated-measures ANOVA evaluated omnibus group, time, and group × time effects within unified models, no formal adjustment for multiple statistical testing was applied. All reported p-values were interpreted within the context of predefined primary and secondary analyses, and corresponding effect sizes and 95% confidence intervals were additionally reported to facilitate interpretation of the precision and magnitude of observed effects.

The primary analysis was predefined according to the intention-to-treat (ITT) principle, whereby all randomized participants were included in the analysis according to their allocated group assignment irrespective of protocol deviations. A per-protocol analysis was also planned for participants achieving ≥80% intervention adherence. As no participant withdrawals, protocol deviations, or missing outcome data occurred during the study, the ITT and per-protocol datasets were identical. In the event of missing outcome data, multiple imputation procedures would have been applied.

Baseline comparability. Between-group differences in continuous baseline variables (age, NPRS, YBT, hop distance, and LSI) were evaluated using independent samples t-tests following verification of normality (Shapiro-Wilk test) and homogeneity of variances (Levene’s test). Categorical variables (gender and affected side) were compared using Fisher’s exact test.

Pre-specification of analyses. The primary prespecified analyses of this randomized controlled trial focused on evaluating the effects of group allocation, time, and group × time interaction on the primary outcome measures (NPRS, Y-Balance Test composite score, and single-hop distance) using repeated-measures ANOVA. These analyses were directly aligned with the primary study hypothesis and sample size estimation. Secondary analyses evaluating limb symmetry index and recovery rate metrics were prespecified supportive analyses intended to characterize multidimensional functional recovery. Correlation analyses, regression modeling, and the exploratory Recovery Efficiency Index were conducted as exploratory analyses intended to generate mechanistic and hypothesis-generating insights rather than definitive causal inferences. Accordingly, these exploratory findings should be interpreted cautiously and validated in larger future studies.

Primary analysis. The effects of group allocation (DPT vs SP), time (T0, T1, T2), and their interaction (group × time) on each primary outcome variable were examined using a two-way mixed-model repeated-measures ANOVA (RM-ANOVA). Mauchly’s test was applied to assess the sphericity assumption, and Greenhouse-Geisser corrections were applied where sphericity was violated. The generalised eta-squared (ηG²) was reported as the measure of effect size, interpreted as small (0.01), medium (0.06), and large (0.14) (Bakeman, 2005).

Correlation analysis. Pearson product-moment correlation coefficients were computed to examine the bivariate associations among change scores for the primary outcome variables (NPRS change, YBT change, hop change), the Recovery Efficiency Index, and the LSI at 12 weeks.

Regression analysis. A multiple linear regression model was constructed to identify independent predictors of improvement in hop performance (dependent variable: change in single-hop distance from T0 to T2). Candidate predictors included group allocation, YBT change, and NPRS change. Unstandardised (B) and standardised (β) regression coefficients, standard errors, t-statistics, 95% confidence intervals, and the overall model fit (R², adjusted R², F-statistic) were reported. Assumptions of linearity, normality of residuals, homoscedasticity, and absence of multicollinearity (variance inflation factor < 5) were verified prior to interpretation. Because balance improvement and hop performance were assessed concurrently at the same post-intervention timepoint, temporal precedence between the variables could not be established. Therefore, the regression analyses were interpreted as associative rather than causal, and no mediation or mechanistic pathway inferences were made.

Between-group comparisons of recovery metrics. Independent samples t-tests were used to compare sport-specific neuromuscular recovery indicators (LSI, recovery rates, and REI) between the DPT and SP groups at 12 weeks. Cohen’s d was computed as the standardised mean difference and interpreted as small (0.20–0.49), medium (0.50–0.79), and large (≥0.80) (Cohen, 1988). Although Cohen’s d values were reported to facilitate standardized comparison between groups, interpretation of treatment effects also considered clinically meaningful improvement thresholds for pain reduction, dynamic balance, hop performance, and limb symmetry restoration in athletic populations. Mean differences with 95% confidence intervals were reported for all between-group comparisons.

## Results

### Participant characteristics and baseline comparability

A total of 60 recreational sprinters diagnosed with patellofemoral pain syndrome were enrolled and randomized equally into the Dynamic Proprioceptive Training (DPT; n = 30) and Strengthening Program (SP; n = 30) groups. All participants completed the 12-week intervention with no dropouts or adverse events recorded. The mean age of participants was 21.37 ± 2.06 years in the DPT group and 21.27 ± 2.13 years in the SP group (p = .854). The gender distribution was identical across groups, with 16 females (53.3%) and 14 males (46.7%) in each arm (p = 1.000, Fisher’s exact test). Similarly, the affected side distribution was balanced, with 18 participants (60.0%) presenting left-sided involvement and 12 (40.0%) right-sided involvement in both groups (p = 1.000).

Independent samples t-tests confirmed that no statistically significant between-group differences existed at baseline for any clinical or functional outcome measure. Specifically, baseline pain intensity (NPRS: DPT, 5.16 ± 0.65 vs. SP, 5.28 ± 0.77; p = .515), dynamic balance (Y-Balance Test: DPT, 87.65 ± 2.18 cm vs. SP, 88.41 ± 2.35 cm; p = .199), single-hop distance on the injured limb (DPT, 442.03 ± 32.95 mm vs. SP, 453.60 ± 33.35 mm; p = .182), single-hop distance on the non-injured limb (DPT, 482.57 ± 33.80 mm vs. SP, 492.63 ± 36.79 mm; p = .275), and limb symmetry index (DPT, 91.58 ± 1.68% vs. SP, 92.10 ± 1.89%; p = .268) were all comparable between groups (Table 2). Although variability in baseline hop distance measurements was moderate, comparable standard deviations were observed across both groups, supporting balanced baseline functional characteristics between interventions.

**Table 2:** Demographic and baseline characteristics of study participants (N = 60).

Variable	DPT Group (n = 30)	SP Group (n = 30)	p-value
**Continuous Variables (Mean ± SD)**			
Age (years)	21.37 ± 2.06	21.27 ± 2.13	0.854
NPRS (0–10)	5.16 ± 0.65	5.28 ± 0.77	0.515
Y-Balance Test (cm)	87.65 ± 2.18	88.41 ± 2.35	0.199
Single-Hop, Injured (mm)	442.03 ± 32.95	453.60 ± 33.35	0.182
Single-Hop, Non-Injured (mm)	482.57 ± 33.80	492.63 ± 36.79	0.275
Limb Symmetry Index (%)	91.58 ± 1.68	92.10 ± 1.89	0.268
**Categorical Variables**			
**Gender, n (%)**			1.000ᵇ
Female	16 (53.3)	16 (53.3)	
Male	14 (46.7)	14 (46.7)	
**Affected Side, n (%)**			1.000ᵇ
Left	18 (60.0)	18 (60.0)	
Right	12 (40.0)	12 (40.0)	

**Note.** DPT = Dynamic Proprioceptive Training; SP = Strengthening Program; NPRS = Numeric Pain Rating Scale; SD = Standard Deviation. ᵃIndependent samples t-test for continuous variables. ᵇFisher’s exact test for categorical variables.

### Changes in clinical and functional outcomes over time

#### Primary outcomes.

**Pain intensity:** Both groups demonstrated significant reductions in pain over the 12-week intervention period (Table 3). In the DPT group, NPRS scores decreased from 5.16 ± 0.65 at baseline to 3.62 ± 0.70 at 6 weeks and 2.18 ± 0.74 at 12 weeks, this represented a 57.8% reduction in pain intensity over the intervention period. The SP group showed a comparatively attenuated trajectory, declining from 5.28 ± 0.77 to 4.27 ± 0.77 and 3.19 ± 0.85 at the respective timepoints. This corresponded to a 39.6% reduction in pain intensity. Repeated-measures ANOVA with a Greenhouse-Geisser correction revealed a significant main effect of time, F(1.64, 95.26) = 1964.95, p < .001, ηG^2^ = .663, indicating a large overall reduction in pain across all participants. A significant main effect of group was also observed, F(1, 58) = 9.91, p = .003, ηG^2^ = .139, reflecting consistently lower pain scores in the DPT group. Critically, a significant group × time interaction emerged, F(1.64, 95.26) = 61.27, p < .001, ηG^2^ = .058, confirming that the rate and magnitude of pain reduction were greater in the DPT group than in the SP group.

**Table 3:** Comparison of clinical and functional outcomes across time points between groups.

Outcome Measure	Group	Baseline (T0)	6 Weeks (T1)	12 Weeks (T2)	Overall Group Difference (95% CI)	Effect	F	p-value	ηG²
**Pain (NPRS)**	DPT (n = 30)	5.16 ± 0.65	3.62 ± 0.70	2.18 ± 0.74	−0.59 [−0.97, −0.22]	Group	9.91	0.003**	0.139
	SP (n = 30)	5.28 ± 0.77	4.27 ± 0.77	3.19 ± 0.85		Time	1964.95	<0.001***	0.663
						Group × Time	61.27	<0.001***	0.058
**Dynamic Balance (Y-Balance)**	DPT (n = 30)	87.65 ± 2.18	95.04 ± 2.12	102.37 ± 2.50	2.53 [1.34, 3.71]	Group	18.18	<0.001***	0.215
	SP (n = 30)	88.41 ± 2.35	92.43 ± 2.57	96.64 ± 2.92		Time	1754.21	<0.001***	0.790
						Group × Time	140.30	<0.001***	0.232
**Hop Performance**	DPT (n = 30)	442.03 ± 32.95	505.62 ± 35.50	569.48 ± 38.34	11.94 [−6.67, 30.54]	Group	1.65	0.204	0.027
	SP (n = 30)	453.60 ± 33.35	494.09 ± 37.69	533.64 ± 42.02		Time	1905.21	<0.001***	0.578
						Group × Time	99.48	<0.001***	0.067

**Note.** Values are presented as Mean ± SD. DPT = Dynamic Proprioceptive Training; SP = Strengthening Program; NPRS = Numeric Pain Rating Scale. Degrees of freedom adjusted using Greenhouse-Geisser correction where sphericity was violated. ges = generalized eta-squared (effect size). **p < 0.01; ***p < 0.001.

**Dynamic balance:** Y-Balance Test composite scores improved progressively in both groups over the intervention period. The DPT group demonstrated improvements from 87.65 ± 2.18 cm at baseline to 95.04 ± 2.12 cm at 6 weeks and 102.37 ± 2.50 cm at 12 weeks, reflecting a total gain of 14.72 cm (16.8% improvement from baseline). The SP group improved from 88.41 ± 2.35 cm to 92.43 ± 2.57 cm and 96.64 ± 2.92 cm, yielding a total gain of 8.23 cm (9.3% improvement from baseline). Repeated-measures ANOVA indicated a significant main effect of time, F(1.14, 66.05) = 1754.21, p < .001, ηG^2^ = .790, representing the largest effect size observed among the assessed outcome domains. The main effect of group was significant, F(1, 58) = 18.18, p < .001, ηG^2^ = .215, and the group × time interaction was also significant, F(1.14, 66.05) = 140.30, p < .001, ηG^2^ = .232, demonstrating that the DPT group achieved substantially greater balance improvements than the SP group across the intervention timeline.

**Fig 2:**
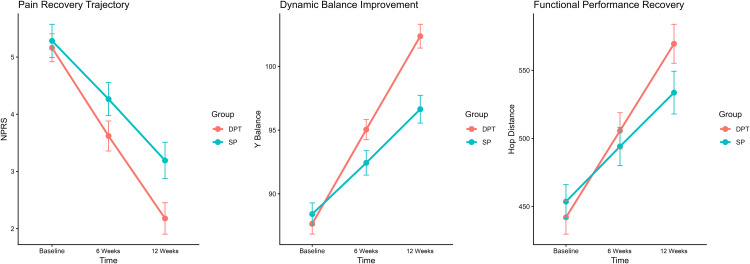
Longitudinal changes in pain, dynamic balance, and functional hop performance following intervention. Mean values with 95% confidence intervals are shown for the DPT and SP groups at baseline, 6 and 12 weeks. Pain decreased and functional performance improved in both groups, with greater improvements in the DPT group. These trends reflect significant time and group × time effects in the repeated measures ANOVA. Error bars indicate 95% confidence intervals.

**Functional hop performance:** Single-hop distance on the injured limb increased in both groups over the 12-week period (Table 3). The DPT group improved from 442.03 ± 32.95 mm to 505.62 ± 35.50 mm at 6 weeks and 569.48 ± 38.34 mm at 12 weeks, representing a total improvement of 127.45 mm, this reflected a 28.8% improvement from baseline.The SP group improved from 453.60 ± 33.35 mm to 494.09 ± 37.69 mm and 533.64 ± 42.02 mm, yielding a gain of 80.04 mm. This corresponded to a 17.6% improvement from baseline. The main effect of time was significant, F(1.17, 67.74) = 1905.21, p < .001, ηG^2^ = .578, confirming robust temporal gains in hop performance across both groups. Notably, the main effect of group was not significant, F(1, 58) = 1.65, p = .204, ηG^2^ = .027, indicating that overall hop distances did not differ significantly between groups when collapsed across time. However, the group × time interaction was significant, F(1.17, 67.74) = 99.48, p < .001, ηG^2^ = .067, indicating that the rate of improvement in hop performance was significantly greater in the DPT group relative to the SP group. The longitudinal trajectories for all three outcome domains are illustrated in Fig 2, which visually depicts the diverging recovery curves between groups across the three assessment timepoints.

#### Secondary outcomes.

**Fig 3:**
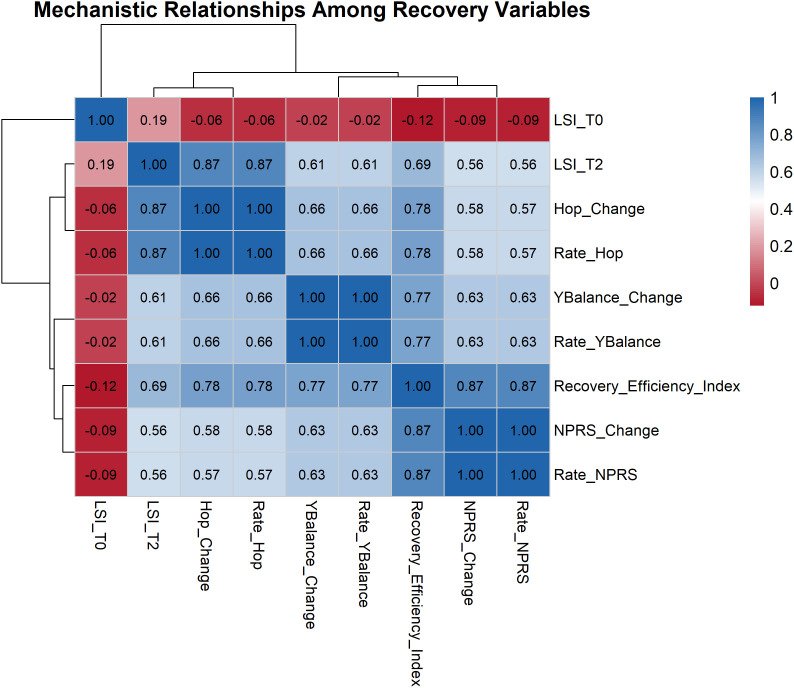
Correlations among clinical and functional recovery variables. Hierarchically clustered Pearson correlation heatmap showing relationships among LSI, hop performance, balance improvement, pain reduction, and recovery efficiency. Strong positive correlations were observed between hop performance and LSI at 12 weeks (r ≈ 0.87). The heatmap demonstrates significant associations among pain reduction, balance improvement, hop performance, and recovery efficiency. All correlations were significant (p < 0.001).

**Associations among clinical and functional recovery variables:** Pearson correlation analysis was performed to examine the bivariate relationships among change scores for the primary outcome variables, the Recovery Efficiency Index (REI), and the limb symmetry index at 12 weeks (LSIT2; Fig 3). All pairwise correlations were positive and statistically significant (p < .001 for all comparisons). Among the primary outcome change scores, the strongest correlation was observed between hop performance change and Y-Balance change (r = .658), followed by the association between NPRS change and Y-Balance change (r = .631), and NPRS change and hop change (r = .575). These moderate-to-strong correlations indicate that improvements in pain, balance, and functional hop performance were interrelated and co-occurred during the recovery process.

The REI demonstrated the highest correlations with NPRS change (r = .870), hop change (r = .781), and Y-Balance change (r = .775), substantiating its role as a composite measure that integrates multidimensional recovery information. The LSI at 12 weeks was most strongly correlated with hop change (r = .871), confirming that limb symmetry restoration was closely linked to functional performance gains on the injured limb. The overall correlation structure among recovery variables is visually represented in the hierarchical cluster heatmap (Fig 3), which delineates distinct clusters of pain-related, balance-related, and performance-related recovery domains while highlighting their interconnectedness through the REI.

### Predictive analyses of functional recovery

#### Predictive regression model.

The following regression-based analyses were exploratory in nature and were not intended to support confirmatory causal conclusions. A multiple linear regression model was constructed to identify independent factors associated with hop performance improvement (Table 4). The overall model was statistically significant, F(3, 56) = 35.91, p < .001, and explained 65.8% of the variance in hop performance change (R^2^ = .658; adjusted R^2^ = .640). Group allocation emerged as the strongest independent factor associated with hop performance improvement (β = −0.98, B = −57.60, SE = 10.54, t = −5.47, p < .001, 95% CI [−78.71, −36.50]), indicating that allocation to the SP group was associated with a 57.60 mm lower improvement in hop performance compared with the DPT group after adjustment for Y-Balance change and NPRS change. Neither Y-Balance change nor NPRS change independently demonstrated significant associations with hop performance improvement after adjustment for group allocation. These findings suggest that the observed between-group differences in hop recovery were more strongly associated with intervention allocation than with the concurrent changes observed in balance or pain outcomes included in the model.

**Table 4:** Multiple linear regression analysis for hop performance recovery.

Variable/ Effect	B/ Estimate	SE	β	t	95% CI	p-value
Intercept	155.92	25.84	—	6.03	[104.16, 207.69]	<0.001***
Y-Balance Change	−0.80	1.14	−0.10	−0.71	[−3.09, 1.48]	0.483
NPRS Change	−5.57	6.17	−0.11	−0.90	[−17.92, 6.78]	0.370
Group (SP vs. DPT)	−57.60	10.54	−0.98	−5.47	[−78.71, −36.50]	<0.001***

**Model Fit:** R² = 0.658, Adjusted R² = 0.640, F(3, 56) = 35.91, p < 0.001.

**Note.** B = unstandardized regression coefficient; SE = standard error; β = standardized coefficient; CI = confidence interval. DPT = Dynamic Proprioceptive Training; SP = Strengthening Program.

#### Sport-specific neuromuscular recovery indicators.

Between-group comparisons of sport-specific neuromuscular recovery indicators are presented in Table 5. At baseline, the limb symmetry index did not differ significantly between groups (DPT, 91.58 ± 1.68% vs. SP, 92.10 ± 1.89%; mean difference [MD] = −0.52%, 95% CI [−1.44, 0.41], t(58) = −1.12, p = .268, d = −0.29). By 12 weeks, however, a pronounced between-group divergence had emerged, with the DPT group achieving a substantially higher LSI (113.68 ± 4.45%) than the SP group (104.45 ± 4.64%), reflecting a mean difference of 9.23% (95% CI [6.88, 11.58], t(58) = 7.86, p < .001, d = 2.03). This large effect size indicates that the DPT intervention produced substantially greater restoration of functional limb symmetry compared with the SP group. However, the post-intervention LSI values exceeding 100% suggest that the injured limb demonstrated hop performance greater than the contralateral limb at 12 weeks. Although this finding may reflect favorable neuromuscular adaptation, enhanced movement confidence, or bilateral training effects, LSI values above 100% should not necessarily be interpreted as optimal symmetry and may also reflect compensatory loading or performance asymmetry. Both groups exceeded the predefined threshold for clinically meaningful pain improvement (≥2-point NPRS reduction) by 12 weeks, while the DPT group demonstrated superior restoration of functional symmetry with post-intervention LSI values substantially exceeding the predefined ≥90% criterion.

**Table 5:** Sport-specific neuromuscular recovery indicators between groups.

Variable	DPT (n = 30) Mean ± SD	SP (n = 30) Mean ± SD	MD	95% CI	t	p-value	Cohen’s d	Effect Size
LSI at Baseline (%)	91.58 ± 1.68	92.10 ± 1.89	−0.52	[−1.44, 0.41]	−1.12	0.268	−0.29	Small
LSI at 12 Weeks (%)	113.68 ± 4.45	104.45 ± 4.64	9.23	[6.88, 11.58]	7.86	<0.001***	2.03	Large
Rate of Pain Recovery (NPRS points/week)	0.25 ± 0.03	0.17 ± 0.04	0.07	[0.06, 0.09]	9.10	<0.001***	2.35	Large
Rate of Balance Recovery (cm/week)	1.23 ± 0.18	0.69 ± 0.16	0.54	[0.45, 0.63]	12.25	<0.001***	3.16	Large
Rate of Hop Recovery (mm/week)	10.62 ± 1.26	6.67 ± 1.66	3.95	[3.19, 4.71]	10.39	<0.001***	2.68	Large
Recovery Efficiency Index (composite)	74.02 ± 8.83	25.98 ± 9.84	48.05	[43.21, 52.88]	19.90	<0.001***	5.14	Large

**Note.** DPT = Dynamic Proprioceptive Training; SP = Strengthening Program; LSI = Limb Symmetry Index; NPRS = Numeric Pain Rating Scale; MD = Mean Difference; CI = Confidence Interval; REI = Recovery Efficiency Index. Independent samples t-test was used for between-group comparisons. Effect sizes interpreted as: small (d = 0.20–0.49), medium (d = 0.50–0.79), large (d ≥ 0.80). ***p < 0.001.

Recovery rate analyses further reinforced the superiority of DPT across all clinical domains. The rate of pain recovery was significantly greater in the DPT group (0.25 ± 0.03 NPRS points/week) compared with the SP group (0.17 ± 0.04 NPRS points/week; MD = 0.08, 95% CI [0.06, 0.09], t(58) = 9.10, p < .001, d = 2.35). The rate of balance recovery favoured the DPT group (1.23 ± 0.18 cm/week vs. 0.69 ± 0.16 cm/week; MD = 0.54, 95% CI [0.45, 0.63], t(58) = 12.26, p < .001, d = 3.17). Similarly, the rate of hop recovery was significantly higher in the DPT group (10.62 ± 1.26 mm/week vs. 6.67 ± 1.66 mm/week; MD = 3.95, 95% CI [3.19, 4.71], t(58) = 10.39, p < .001, d = 2.68). All three recovery rate comparisons yielded large effect sizes (d > 2.0), indicating substantial between-group differences that exceeded conventional thresholds for clinical significance.

The exploratory composite Recovery Efficiency Index (REI), which was designed to summarize multidimensional recovery patterns across outcome domains, was higher in the DPT group (74.02 ± 8.83) compared with the SP group (25.98 ± 9.84; MD = 48.05, 95% CI [43.21, 52.88], t(58) = 19.90, p < .001, d = 5.14). This represented the largest effect size observed across all comparisons in the study, signifying that the DPT intervention produced a substantially more efficient overall recovery profile than the conventional SP.

#### Multidomain recovery trajectories.

**Fig 4:**
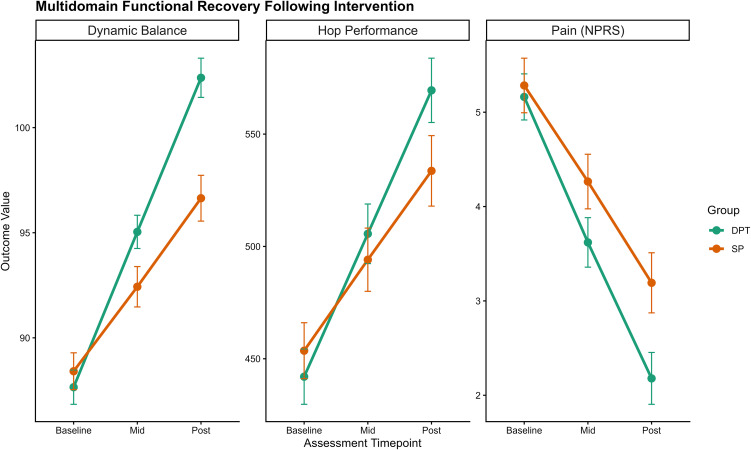
Multidomain functional recovery trajectories across the intervention period. Faceted visualization showing changes in dynamic balance (Y-Balance Test), hop performance (single-hop distance), and pain intensity (NPRS) across baseline, mid-, and post-intervention. Mean values with 95% confidence intervals are presented for the Dynamic Proprioceptive Training (DPT) and Strengthening Program (SP) groups. The DPT group showed greater improvements in balance and hop performance, along with larger pain reduction, highlighting multidimensional functional recovery during rehabilitation.

The multidomain functional recovery trajectories for pain, dynamic balance, and hop performance are presented in Fig 4. Visual inspection of the group-specific recovery curves reveals a consistent pattern across all three domains: both groups improved over time, but the DPT group demonstrated steeper recovery gradients with progressively widening between-group separations from baseline through the 12-week endpoint. The divergence between groups became most apparent between 6 and 12 weeks, suggesting that the observed improvements associated with dynamic proprioceptive training continued to accrue beyond the initial phase of rehabilitation. Taken together with the significant group × time interactions reported in Table 3 and the large effect sizes documented in Table 5, these trajectories provide convergent evidence that DPT confers a multidimensional advantage over conventional strengthening in the rehabilitation of recreational sprinters with patellofemoral pain syndrome.

## Discussion

### Principal findings

This randomized controlled trial examined the effectiveness of a 12-week dynamic proprioceptive training program compared with a conventional progressive strengthening program in recreational sprinters with patellofemoral pain syndrome (PFPS). Both interventions resulted in significant improvements in pain intensity, dynamic balance, and functional hop performance over the intervention period. However, the dynamic proprioceptive training program produced consistently greater improvements across all outcome domains.

Participants in the proprioceptive training group demonstrated larger reductions in pain, greater improvements in dynamic balance, and superior gains in functional hop performance compared with those receiving strengthening alone. Additionally, multidimensional recovery indicators including limb symmetry index and the composite Recovery Efficiency Index showed substantially greater improvements in the proprioceptive training group. Collectively, these findings suggest that interventions targeting sensorimotor control and reactive neuromuscular function may enhance rehabilitation outcomes beyond those achieved through conventional strengthening programs in athletic populations with PFPS.

### Potential explanations for the observed training effects

The stronger group effect observed in the regression analysis suggests that hop performance improvement was more strongly associated with intervention allocation than with the concurrent changes observed in pain or balance measures included in the model. The superior outcomes observed following dynamic proprioceptive training are associated with the greater neuromuscular demands imposed by this intervention. Although the precise mechanisms underlying this association cannot be determined from the present data, the findings may reflect multidimensional neuromuscular and task-specific adaptations not fully captured by the measured clinical variables [36,37]. Potential contributing factors may include improved quadriceps activation and lower-limb neuromuscular coordination, enhanced movement confidence following pain reduction, altered movement strategies during dynamic tasks, or task-specific motor adaptations associated with repeated proprioceptive challenge [38–40]. These tasks may also promote sensorimotor integration and improved neuromuscular coordination during functional movement [38–40]. However, because electromyographic, kinetic, and biomechanical assessments were not performed, these proposed mechanisms should be interpreted as hypothesis-generating explanations rather than directly demonstrated effects.

Dynamic proprioceptive training also requires athletes to adapt rapidly to changes in body position and external perturbations [40,41]. These task demands improve both anticipatory and reactive motor control strategies, which are critical for maintaining lower-limb alignment during high-velocity athletic movements [42]. However, because electromyographic and biomechanical assessments were not performed in the present study, these proposed mechanisms should be interpreted as hypothesis-generating explanations rather than directly demonstrated effects [43,44].

Another contributing factor exhibit the task-specific nature of the proprioceptive exercises used in the present study [45,46]. The training program incorporated movements that closely resembled the neuromuscular demands of sprinting, including single-leg landings, reactive directional movements, and multidirectional reach tasks [47,48]. According to the principle of training specificity, exercises that replicate the biomechanical demands of sport are more likely to produce transferable improvements in functional performance [46,49].

The higher limb symmetry index observed in the proprioceptive training group reflects improved functional capacity of the affected limb relative to baseline levels [50–52]. The elevated post-intervention limb symmetry values (>100%) observed in the DPT group indicate that hop performance of the previously symptomatic limb exceeded that of the contralateral limb at 12 weeks, a phenomenon previously reported following targeted neuromuscular rehabilitation [53–55]. This finding may reflect neuromuscular adaptation, improved movement confidence following pain reduction, enhanced neuromuscular coordination, task-specific training effects, or bilateral adaptations resulting from unilateral training stimuli [53–55]. However, LSI values exceeding 100% should be interpreted cautiously, as they do not necessarily represent ideal functional symmetry and may also reflect compensatory movement strategies or residual asymmetry in limb loading patterns. Because biomechanical analyses were not performed, the present study cannot determine whether the observed values reflected beneficial functional overcompensation or undesirable compensatory mechanics. Nevertheless, these findings suggest that dynamic proprioceptive training may facilitate restoration of functional symmetry during athletic tasks [19,56,57].

The absence of a significant mediation effect of dynamic balance on hop performance improvement suggests that additional mechanisms may contribute to functional recovery following dynamic proprioceptive training. Potential contributing factors may include improved movement confidence, reduced fear of movement, enhanced sensorimotor integration, altered neuromuscular coordination, or localized proprioceptive acuity independent of global balance improvements. Similar multidimensional rehabilitation mechanisms involving both sensorimotor and psychological adaptation have previously been proposed in mindfulness-based rehabilitation models for patellofemoral pain syndrome [58]. Accordingly, the functional improvements observed in the present study may reflect broader neuromuscular and behavioral adaptations beyond improvements in dynamic balance alone. However, because psychological, electromyographic, and biomechanical variables were not directly assessed in the present study, these explanations should be interpreted as hypothesis-generating rather than confirmed mechanistic findings.

### Contextualizing the findings within existing evidence

The present findings are consistent with previous studies reporting beneficial effects of neuromuscular and proprioceptive training in individuals with PFPS. Prior research has shown that rehabilitation programs combining strengthening with sensorimotor training can produce greater improvements in pain and functional outcomes compared with strengthening alone [32,59]. For example, previous studies have reported improved balance, proprioception, and functional performance when proprioceptive exercises are incorporated into PFPS rehabilitation protocols [19,28,29,60].

The present study extends this body of evidence in several important ways. First, it specifically examined a sport-relevant population of recreational sprinters, whose functional demands differ from those of general or sedentary populations commonly studied in PFPS research. Second, the study incorporated a structured proprioceptive training protocol emphasizing perturbation-based tasks and sport-specific movement patterns, which may be particularly relevant for athletes engaged in high-velocity activities. Third, the use of multiple functional outcome measures enabled evaluation of recovery across pain, balance, and performance domains.

Importantly, although the proprioceptive intervention produced superior outcomes, the strengthening program also resulted in meaningful clinical improvements. This observation aligns with the extensive evidence supporting hip and knee strengthening as an effective treatment for PFPS. Therefore, strengthening remains a valid component of rehabilitation programs, while the addition of proprioceptive training may further enhance functional recovery.

### Clinical translation for athletic rehabilitation

The findings of this study have several practical implications for rehabilitation of athletes with PFPS. First, incorporating dynamic proprioceptive exercises into rehabilitation programs may improve functional outcomes in athletes who require high levels of reactive neuromuscular control [61–63]. Exercises involving unstable surfaces, perturbation challenges, and sport-specific movement patterns may help restore dynamic stability during athletic tasks [64–66]. Importantly, the pain reduction observed in the DPT group exceeded the commonly accepted 2-point clinically important difference for the NPRS. Improvements in hop performance and limb symmetry additionally suggest clinically relevant restoration of sport-specific lower-limb function in recreational sprinters with PFPS. Second, the greater recovery rates observed in the proprioceptive training group suggest that such interventions may accelerate functional improvements [67–69]. Faster recovery of balance and performance capacity may facilitate earlier return to sport participation, which is particularly relevant for competitive athletes [70,71]. Although the dynamic proprioceptive training program required additional unstable-surface equipment and greater therapist supervision during progression, the exercises were implemented using relatively low-cost and widely available rehabilitation tools such as foam pads, BOSU balls, and wobble boards. The greater functional improvements and accelerated recovery observed in the DPT group may therefore justify the modest increase in rehabilitation complexity, particularly in athletic populations requiring rapid restoration of sport-specific neuromuscular control.

Third, assessment of functional symmetry using measures such as the limb symmetry index may provide useful information for evaluating readiness to resume athletic activity [72,73]. Rehabilitation programs that integrate strength training with neuromuscular and proprioceptive exercises may therefore provide a more comprehensive strategy for restoring sport-specific function [74,75]. Finally, the multidimensional assessment approach used in this study highlights the importance of evaluating rehabilitation outcomes beyond pain reduction alone [76–78]. Functional performance and neuromuscular control measures may provide valuable insights into recovery of athletic capacity [79,80].

### Strengths, limitations, and methodological considerations

This study has several methodological strengths. The randomized controlled trial design with assessor blinding reduced the risk of selection and measurement bias. Standardized intervention protocols and treatment fidelity procedures ensured consistency in program delivery. Additionally, validated outcome measures were used to assess pain, dynamic balance, and functional performance, enhancing the reliability of the findings.

Several limitations should also be considered. First, the study was conducted at a single center with a relatively homogeneous sample restricted to recreational sprinters aged 18–30 years with unilateral patellofemoral pain syndrome, which may limit generalizability to elite athletes, older individuals, younger adolescents, sedentary populations, or patients presenting with bilateral symptoms and differing functional demands. Because sprint athletes exhibit unique neuromuscular and biomechanical requirements, the observed intervention effects may not directly translate to other athletic or non-athletic populations. The relatively large variability observed in baseline hop performance measures may also have reduced statistical precision and affected the power to detect smaller between-group differences in functional performance outcomes. Furthermore, the use of a fixed block size of four during randomization may have increased the theoretical risk of allocation predictability, although allocation concealment using sequentially numbered opaque sealed envelopes was implemented to minimize selection bias Although both intervention groups received equivalent session frequency and overall treatment duration, the exact muscular workload and total contraction volume between the dynamic proprioceptive training and strengthening interventions were not directly quantified. Differences in exercise modality, neuromuscular demand, and mechanical work may therefore have contributed to the observed outcomes and should be considered when interpreting the findings. Future studies should incorporate objective workload monitoring to better standardize treatment volume across intervention groups.

Second, participants and treating therapists could not be blinded to the intervention because of the nature of the rehabilitation procedures, which may have introduced performance bias. Third, the 12-week intervention period and absence of longer-term follow-up assessments did not permit evaluation of the durability or long-term stability of the observed treatment effects, including symptom recurrence, sustained functional recovery, or return-to-sport performance. Given the chronic and recurrent nature of patellofemoral pain syndrome, it remains unclear whether the improvements observed at 12 weeks would be maintained over longer follow-up periods such as 6 or 12 months.

Although the Recovery Efficiency Index (REI) provided an integrated representation of multidimensional rehabilitation recovery, the index was exploratory in nature and has not yet undergone external validation or psychometric evaluation. Furthermore, the absence of established clinical thresholds limits interpretation of what constitutes clinically meaningful recovery based on REI values alone. Importantly, separate outcome measures remain clinically essential because improvements in pain, balance, and functional performance may not always occur proportionally within individual patients. Accordingly, the REI should be interpreted as a complementary exploratory summary measure rather than a replacement for domain-specific clinical assessment.

In addition, several secondary statistical procedures, including correlation analyses, regression modeling, and exploratory composite recovery metrics, were performed in a relatively modest sample. Although these analyses were prespecified as exploratory and interpreted cautiously, the study was not specifically powered for complex predictive modeling. Therefore, these findings should be considered hypothesis-generating and require confirmation in larger adequately powered studies.

Biomechanical and neuromuscular assessments such as motion analysis or electromyography were not included, limiting the ability to directly examine changes in movement mechanics. This limitation is particularly relevant when interpreting the elevated post-intervention LSI values observed in the DPT group, as movement quality and compensatory loading strategies could not be directly evaluated. Psychological factors such as fear of movement or pain-related beliefs were also not assessed, although these factors may influence rehabilitation outcomes. Furthermore, the absence of a non-intervention, sham, or placebo control group prevents conclusions regarding the natural course of PFPS recovery and limits the ability to determine the extent to which nonspecific treatment effects, participant expectations, therapist interaction, or attention-related effects may have contributed to the observed improvements. In addition, although group allocation independently predicted hop performance improvement, the study design and concurrent measurement of balance and hop performance outcomes do not permit determination of temporal sequencing or mechanistic pathways underlying the observed intervention effect. Because these variables were assessed at the same post-intervention timepoint, causal mediation relationships could not be established, and the observed associations should therefore be interpreted cautiously. Finally, individual directional Y-Balance reach distances were not retained for separate analysis because the normalized composite score was prespecified as the primary balance outcome. Consequently, direct directional comparisons with studies reporting anterior, posteromedial, and posterolateral reach distances individually were limited.

### Directions for future research

Future research should investigate the long-term effectiveness of proprioceptive training programs and their impact on return-to-sport outcomes. Incorporating biomechanical assessments, including three-dimensional motion analysis and electromyographic evaluation, would help clarify the movement adaptations associated with these interventions. Multicenter trials involving diverse athletic populations are needed to strengthen the generalizability of the findings, while studies exploring individualized rehabilitation approaches based on baseline neuromuscular deficits may further improve treatment effectiveness. Additional investigations should also examine optimal training parameters, including the frequency, intensity, and progression of proprioceptive exercises, to inform evidence-based rehabilitation protocols for PFPS. Furthermore, future studies should evaluate the psychometric properties, weighting strategies, external validity, and clinical interpretability of composite multidomain recovery indices such as the Recovery Efficiency Index. Randomized controlled trials integrating proprioceptive rehabilitation with cognitive-behavioral or mindfulness-based strategies may also help optimize multidimensional recovery and return-to-sport outcomes in athletes with patellofemoral pain syndrome.

## Supporting information

S1 File Protocol. (DOCX)

S2 File CONSORT checklist. (DOCX)
