# Supplementary material for: Dynamic proprioceptive training improves functional recovery in sprinters with patellofemoral pain: A randomized trial
Source: PLoS One. 2026 Jul 24;21(7):e0353107. doi: 10.1371/journal.pone.0353107 (PMC13399522; doi:10.1371/journal.pone.0353107)
Supplement: S1 File — (DOCX) [file pone.0353107.s001.docx]

**S1 File Protocol: Multidomain Functional Recovery Following Dynamic Proprioceptive Training in Sprinters with Patellofemoral Pain: A Randomized Controlled Trial**

**Background and Rationale**

Patellofemoral pain syndrome (PFPS) is a prevalent musculoskeletal condition among physically active individuals, particularly runners and sprinters, characterized by anterior knee pain and functional limitations. Emerging evidence suggests that, in addition to muscular weakness, deficits in proprioception and dynamic postural control contribute significantly to symptom persistence and impaired performance. Conventional rehabilitation strategies primarily emphasize strengthening; however, these approaches may not sufficiently address sensorimotor impairments.

Dynamic proprioceptive training, incorporating unstable surfaces and perturbation-based tasks, has been proposed as a strategy to enhance neuromuscular coordination and sport-specific functional recovery. Despite its growing use, high-quality randomized controlled trials comparing its effectiveness against conventional strengthening in athletic populations remain limited. This study was designed to address this gap.

### ****Objectives and Hypotheses****

#### **Primary Objective**

The primary objective of this randomized controlled trial is to rigorously compare the effectiveness of a structured dynamic proprioceptive training program versus a conventional progressive strengthening program in improving multidimensional functional outcomes among recreational sprinters with patellofemoral pain syndrome. Specifically, the study aims to evaluate between-group differences over time in:

- Pain intensity, assessed using the Numeric Pain Rating Scale (NPRS), reflecting symptomatic relief
- Dynamic balance, quantified using the Y-Balance Test, representing sensorimotor control and postural stability
- Functional hop performance, measured via single-leg hop distance, reflecting lower extremity functional capacity and sport-specific performance

#### **Secondary Objectives**

The secondary objectives of this study are to provide a comprehensive evaluation of functional recovery and to explore underlying mechanisms associated with rehabilitation outcomes:

- To assess restoration of interlimb functional symmetry using the Limb Symmetry Index (LSI), as an indicator of readiness for return to sport
- To quantify domain-specific recovery rates (pain reduction, balance improvement, and functional performance gains) to evaluate the temporal dynamics and efficiency of rehabilitation
- To develop and analyze a composite Recovery Efficiency Index (REI), integrating multidimensional improvements into a unified measure of overall rehabilitation effectiveness
- To investigate the interrelationships among changes in pain, dynamic balance, and functional performance using correlation analyses
- To examine whether improvements in dynamic balance mediate the effect of intervention type on functional performance outcomes through causal mediation analysis
- To identify independent predictors of functional recovery, particularly improvements in hop performance, using multivariable regression modelling

### ****Hypotheses****

#### **Primary Hypotheses**

**Null Hypothesis (H₀₁):** There is no significant difference between dynamic proprioceptive training and conventional strengthening in improving pain intensity, dynamic balance, and functional hop performance among recreational sprinters with patellofemoral pain syndrome.

**Alternative Hypothesis (H₁₁):** Dynamic proprioceptive training produces significantly greater improvements in pain intensity, dynamic balance, and functional hop performance compared with conventional strengthening.

#### **Secondary Hypotheses**

**Limb Symmetry and Recovery Efficiency**

**H₀₂:**
There is no significant difference between groups in limb symmetry restoration or recovery efficiency.

**H₁₂:**
Dynamic proprioceptive training results in significantly greater limb symmetry restoration and higher recovery efficiency compared with conventional strengthening.

**Association Between Variables**

**H₀₃:**
There is no significant association between improvements in dynamic balance and functional performance.

**H₁₃:**
Improvements in dynamic balance are significantly associated with gains in functional performance.

**Mediation Effect**

**H₀₄:**
Dynamic balance does not mediate the relationship between intervention type and functional hop performance.

**H₁₄:**
Dynamic balance mediates the relationship between intervention type and functional hop performance.

**Predictive Model**

**H₀₅:**
Group allocation is not a significant independent predictor of functional recovery outcomes.

**H₁₅:**
Group allocation is a significant independent predictor of functional recovery outcomes.

#### **Overall Hypothesis**

**H₀₆:**
Dynamic proprioceptive training does not produce significantly greater multidomain functional recovery compared with conventional strengthening.

**H₁₆:**
Dynamic proprioceptive training produces significantly greater multidomain functional recovery compared with conventional strengthening.

**Study Design**

This study was a two-arm, parallel-group, assessor-blinded randomized controlled trial with a 1:1 allocation ratio. A repeated-measures design was employed with three timepoints:

- Baseline (T0)
- Mid-intervention (6 weeks, T1)
- Post-intervention (12 weeks, T2)

The study was conducted in accordance with CONSORT guidelines.

**Study Setting**

The trial was conducted at the outpatient physiotherapy rehabilitation unit of Saveetha College of Physiotherapy, Saveetha Institute of Medical and Technical Sciences (SIMATS), Chennai, India, between August 2025 and December 2025. All assessments and interventions were performed in a standardized, controlled clinical environment.

### ****Participants****

Participants comprised recreational sprinters diagnosed with unilateral patellofemoral pain syndrome (PFPS), recruited through convenience sampling from university sports teams, local athletic clubs, and community rehabilitation centers.

#### **Inclusion Criteria**

Participants were eligible for inclusion if they met all of the following criteria:

- Age between 18 and 30 years
- Clinically confirmed diagnosis of unilateral patellofemoral pain syndrome, characterized by anterior or retropatellar knee pain aggravated by functional activities such as squatting, stair climbing, running, or prolonged sitting
- Pain intensity of ≥3 on the Numeric Pain Rating Scale (NPRS) during at least one aggravating activity
- Regular participation in sprint-based activities (≥3 sessions per week) for at least six months prior to enrollment
- Symptom duration of at least four weeks
- Willingness to participate in the 12-week intervention and comply with all assessment procedures

#### **Exclusion Criteria**

Participants were excluded if they met any of the following conditions:

- History of knee surgery or significant ligamentous injury (e.g., anterior cruciate ligament, posterior cruciate ligament, or collateral ligament injury)
- Presence of meniscal pathology, patellar instability, or recurrent dislocation/subluxation
- Neurological disorders, systemic musculoskeletal conditions, or inflammatory diseases affecting lower limb function
- Use of analgesic, corticosteroid, or other medications that could influence pain perception within two weeks prior to enrollment
- Participation in any concurrent structured rehabilitation, physiotherapy, or strength training program targeting the lower extremity
- Any other condition that, in the opinion of the investigators, could interfere with safe participation or outcome assessment

### Sample Size Estimation

An a priori sample size estimation was performed using G*Power software (version 3.1.9.7; Heinrich-Heine-Universität Düsseldorf, Germany) based on a repeated measures analysis of variance (ANOVA) design with within–between interaction effects.

The following parameters were specified: a medium-to-large effect size (f = 0.25), an alpha level (α) of 0.05, a statistical power (1 − β) of 0.80, two intervention groups, and three repeated measurement timepoints (baseline, 6 weeks, and 12 weeks). A correlation among repeated measures of 0.50 and a nonsphericity correction (ε) of 1 were assumed.

The analysis indicated that a minimum total sample size of 44 participants would be required to detect a statistically significant group × time interaction effect.

To account for potential attrition and ensure adequate statistical power, the sample size was increased by approximately 25%, resulting in a final target sample of 60 participants, with 30 participants allocated to each group.

### ****Randomisation and Allocation Concealment****

Participants who met eligibility criteria and provided informed consent were randomly allocated to either the Dynamic Proprioceptive Training (DPT) group or the Strengthening Program (SP) group in a 1:1 ratio. Randomisation was performed using a computer-generated block randomisation sequence with a fixed block size of four, prepared by an independent statistician who was not involved in participant recruitment, assessment, or intervention delivery.

Allocation concealment was ensured through the use of sequentially numbered, opaque, sealed envelopes. Each envelope contained the group assignment and was opened only after completion of baseline assessment, thereby preventing selection bias and ensuring that allocation remained concealed from both participants and assessors at the time of enrolment.

### ****Blinding****

Due to the nature of the physical interventions, blinding of participants and treating physiotherapists was not feasible. However, all outcome assessments were conducted by trained assessors who were blinded to group allocation throughout the study period. In addition, the statistician responsible for data analysis remained blinded to group allocation until completion of the primary analyses.

Participants were instructed not to disclose their group allocation to the assessors to minimize the risk of detection bias.

### ****Interventions****

Both intervention groups received supervised rehabilitation sessions administered by licensed physiotherapists with experience in musculoskeletal rehabilitation. All sessions were conducted on a one-to-one basis to ensure treatment fidelity and adherence.

#### **Dynamic Proprioceptive Training (DPT)**

Participants in the DPT group underwent a structured, progressive neuromuscular training program designed to enhance sensorimotor control, dynamic joint stability, and sport-specific functional performance. The program included:

- Exercises performed on unstable surfaces (e.g., foam pads, BOSU balls, wobble boards) to challenge postural control
- Perturbation-based balance training involving external and self-induced disturbances
- Reactive stepping and agility drills incorporating multidirectional movement patterns
- Single-leg functional tasks and sport-specific activities, including hop-and-hold landings and dynamic reach tasks

Exercise progression was individualized and achieved by systematically increasing task complexity, reducing base of support, increasing surface instability, and incorporating higher-velocity sport-specific movements.

#### **Strengthening Program (SP)**

Participants in the SP group received a conventional progressive strengthening program targeting key muscle groups implicated in PFPS. The program included:

- Quadriceps strengthening, with emphasis on the vastus medialis obliquus
- Hip abductor and external rotator strengthening
- Core stabilization exercises

Exercises were performed using both open and closed kinetic chain approaches, including isometric and isotonic contractions. Resistance intensity was progressively increased from approximately 60% to 80% of one-repetition maximum (1RM), with adjustments based on individual tolerance and performance.

#### **Intervention Schedule**

- Frequency: Three sessions per week
- Total duration: 12 weeks
- Session duration: 45–60 minutes

Participants were instructed to maintain their usual daily activities but refrain from engaging in additional structured rehabilitation or training programs during the study period.

### ****Outcome Measures****

All outcome measures were assessed at three standardized timepoints: baseline (T0), mid-intervention (6 weeks, T1), and post-intervention (12 weeks, T2), by blinded assessors using standardized protocols.

#### **Primary Outcomes**

- **Pain Intensity:** Assessed using the Numeric Pain Rating Scale (NPRS), an 11-point scale ranging from 0 (no pain) to 10 (worst imaginable pain)
- **Dynamic Balance:** Assessed using the Y-Balance Test, with composite reach distance normalized to limb length
- **Functional Performance:** Assessed using the single-leg hop for distance test on the affected limb

#### **Secondary Outcomes**

- **Limb Symmetry Index (LSI):** Calculated as the percentage ratio of hop distance between the affected and unaffected limbs
- **Recovery Rates:** Computed as the rate of change in pain, balance, and functional performance over the intervention period
- **Recovery Efficiency Index (REI):** A composite metric derived from standardized recovery rates across multiple domains, reflecting overall rehabilitation efficiency

### ****Data Collection and Standardization****

All outcome assessments were conducted using standardized and reproducible protocols to minimize measurement variability and ensure internal validity. The following procedures were implemented:

- All assessments were performed by the same trained and blinded assessor across all timepoints to reduce inter-rater variability
- A standardized warm-up protocol (5-minute low-intensity cycling) was administered prior to functional testing
- The sequence of outcome assessment was kept consistent across sessions (pain assessment followed by balance testing and then hop performance) to control for fatigue effects
- Identical equipment and measurement tools were used for all participants at each timepoint
- Assessments were conducted at a similar time of day (±2 hours) to minimize diurnal variation

### ****Statistical Analysis****

All statistical analyses were performed using R software (version 4.3.0; R Foundation for Statistical Computing, Vienna, Austria). Statistical significance was set at a two-tailed α level of 0.05.

Descriptive statistics were expressed as mean ± standard deviation for continuous variables and as frequencies with percentages for categorical variables.

The primary analysis was conducted using mixed-model repeated measures analysis of variance (RM-ANOVA) to evaluate the effects of group (DPT vs. SP), time (T0, T1, T2), and their interaction (group × time) on outcome variables. Assumptions of normality and sphericity were assessed using the Shapiro–Wilk test and Mauchly’s test, respectively, with Greenhouse–Geisser corrections applied where necessary.

Effect sizes were reported using generalized eta-squared (η²) for ANOVA and Cohen’s d for between-group comparisons.

Pearson correlation analysis was conducted to assess associations among changes in primary outcomes and composite recovery measures.

Causal mediation analysis was performed using a bootstrap resampling approach (1,000 simulations) to evaluate whether dynamic balance mediated the relationship between intervention type and functional performance outcomes.

Multiple linear regression analysis was conducted to identify independent predictors of functional recovery, with appropriate checks for multicollinearity, normality of residuals, and homoscedasticity.

### ****Handling of Missing Data****

All randomized participants completed the study and were included in the final analysis. As no missing data were observed, no imputation methods were required.

### ****Adherence and Fidelity****

Participant adherence to the intervention protocol was monitored using session attendance logs maintained by the treating physiotherapists. A minimum attendance threshold of 80% (≥29 out of 36 sessions) was required for adherence.

Intervention fidelity was ensured through:

- Use of standardized, pre-defined intervention protocols
- Training of physiotherapists prior to study initiation
- Periodic monitoring and verification of intervention delivery

### ****Safety and Adverse Events****

Participants were monitored throughout the intervention period for any adverse events or symptom exacerbations. All events were documented using standardized reporting procedures.

No adverse events or serious complications were reported during the study.

### ****Ethical Considerations****

This study was approved by the Institutional Scientific Review Board of Saveetha College of Physiotherapy, Saveetha Institute of Medical and Technical Sciences (SIMATS), India, prior to participant recruitment.

The study was conducted in accordance with the ethical principles outlined in the Declaration of Helsinki. Written informed consent was obtained from all participants prior to enrollment, and participants were informed of their right to withdraw at any time without penalty.

Confidentiality and anonymity of participant data were maintained throughout the study.

### ****Trial Registration****

The trial was prospectively registered with the Clinical Trials Registry of India (CTRI/2025/07/090253), a primary registry of the World Health Organization’s International Clinical Trials Registry Platform.

### ****Data Availability****

De-identified individual participant data, statistical code, and data dictionary supporting the findings of this study are available from the corresponding author upon reasonable request.
